# Biological and Clinical Significance of the CCR5/CCL5 Axis in Hepatocellular Carcinoma

**DOI:** 10.3390/cancers12040883

**Published:** 2020-04-05

**Authors:** Santosh K. Singh, Manoj K. Mishra, Brian M. Rivers, Jennifer B. Gordetsky, Sejong Bae, Rajesh Singh

**Affiliations:** 1 Department of Microbiology, Biochemistry and Immunology, Cancer Health Equity Institute, Morehouse School of Medicine, Atlanta, GA 30310, USA; sksingh@msm.edu; 2 Department of Biological Sciences, Alabama State University, Montgomery, AL 36101, USA; mmishra@alasu.edu; 3 Cancer Health Equity Institute, Morehouse School of Medicine, Atlanta, GA 30310, USA; brivers@msm.edu; 4 Departments of Pathology and Urology, Vanderbilt University Medical Center, Nashville, TN 37232, USA; Jennifer.b.gordetsky@vumc.org; 5 Department of Medicine, University of Alabama at Birmingham School of Medicine, Birmingham, AL 35205, USA; bsejong@uab.edu

**Keywords:** hepatocellular carcinoma, CCR5, CCL5, maraviroc, EMT

## Abstract

Despite the improvement in survival for patients with liver cancer (LCa) in recent decades, only one in five patients survive for 5 years after diagnosis. Thus, there is an urgent need to find new treatment options to improve patient survival. For various cancers, including LCa, the chemokine CCL5 (RANTES) facilitates tumor progression and metastasis. Since the function of the CCR5/CCL5 interaction in LCa cell proliferation and migration is poorly understood, the present study was undertaken to investigate the role of the CCR5/CCL5 axis in these processes. Flow cytometry, RT-PCR, Western blot, and immunofluorescence techniques were used to quantify the expression of CCR5 and CCL5 in LCa cells. To determine the biological significance of CCR5 expressed by LCa cell lines, a tissue microarray of LCas stained for CCR5 and CCL5 was analyzed. The results showed higher expression (*p* < 0.001) of CCR5 and CCL5 in hepatocellular carcinoma (HCC) tissues compared to non-neoplastic liver tissues. Furthermore, to delineate the role of the CCR5/CCL5 interaction in LCa cell proliferation and migration, various LCa cells were treated with maraviroc, a CCR5 antagonist, in the presence of CCL5. These data demonstrated the biological and clinical significance of the CCR5/CCL5 axis in LCa progression. The targeting of this axis is a promising avenue for the treatment of LCa.

## 1. Introduction

Liver cancer (LCa) is the seventh leading cause of cancer-related mortality worldwide, and hepatocellular carcinoma (HCC) accounts for 75–85% among all the types of LCas [1]. In the western world, HCC primarily affects patients with cirrhosis, secondary to hepatitis C virus (HCV) infection or alcoholism. In other parts of the world, HCC is associated with hepatitis B virus (HBV) infections [2,3]. Patients with HCC exhibit a poor prognosis, with overall median survival of 11 months, and an all-inclusive 1-year survival rate of less than 50% [4]. Early HCCs are well differentiated, <2 cm in size, with poorly defined margins and vague nodules; progressed HCCs are >2 cm in size, with moderate differentiation and a distinct nodular type [5]. For patients with advanced HCCs, while standard chemotherapy is ineffective; other treatment modalities include trans-arterial chemoembolization (TACE), ethanol injection, radio-frequency or microwave ablation, and selective internal radiation therapy (SIRT) [6,7,8]. However, there is no efficient treatment currently available, except for liver resection and liver transplantation, which provide a 5-year survival rate of up to 70% [4]. In the development and progression of HCCs, chemokines and their interactions with receptors influence the processes by allowing tumor cells to evade the immune system and by promoting inflammation, angiogenesis, and metastasis.

Chemokines, a family of small (8–14 kDa) chemotactic molecules present within the lymph system and various tissues, orchestrate the migration of immune cells during evasion of the immune response [9]. They are categorized into four families, namely CXC, CC, CX3C, and XC according to the presence of four cysteine residues in conserved locations [10]. Originally, chemokines were regarded for their role in interacting with their cognate receptors, mostly present in neutrophils, lymphocytes, endothelial cells, and epithelial cells [11,12]. However, in recent decades, the role of chemokines and their receptors in promoting growth, progression, and metastasis of tumors has been established [13,14].

With the discovery of the functions of chemokines and their receptors in various cancers, their roles in HCC have been investigated. These include evaluation of the CXCL12/CXCR4 axis in angiogenesis [15], the CCL20/CCR6 axis in the growth of the hepatoma cell line Huh7 [16]; and the CCL5/CCR1 axis in promoting the metastasis and invasion of HCC cells [17]. The chemokine ligand CCL5/RANTES (regulated upon activation, normal T-cell-expressed and secreted) promotes carcinogenesis and stroma genesis [18]. CCL5, a target gene of a nuclear factor kappa-light-chain-enhancer of activated B cells (NF-κB) [19], is expressed by various types of cells (T lymphocytes, macrophages, platelets, synovial fibroblasts, and tubular epithelium) [20]. CCL5 is secreted by certain types of tumor cells [21], and clinical studies show that elevated levels of CCL5 in tissues and plasma reflect adverse conditions for patients with melanoma, breast, cervical, prostate, gastric, or pancreatic cancer [22]. Although CCL5 binds to CCR1 and CCR3 receptors, the activity of CCL5 is expressed by binding to CCR5 [18]. 

CCL5 and CCR5 are involved in the growth, progression, and metastasis of various cancers [20]. However, their significance in HCC is not well studied. Thus, the present study sought to understand the clinicopathological and prognostic significance of CCL5 and CCR5 in HCCs. We examined HCC clinical specimens and HCC cell lines and showed that CCL5 and its receptor were over-expressed in primary LCas and that these factors are involved in LCa progression and metastasis.

## 2. Results

### 2.1. A Microarray of Human HCC Tissues Shows Upregulated CCR5 and CCL5 Levels

**Figure 1:**
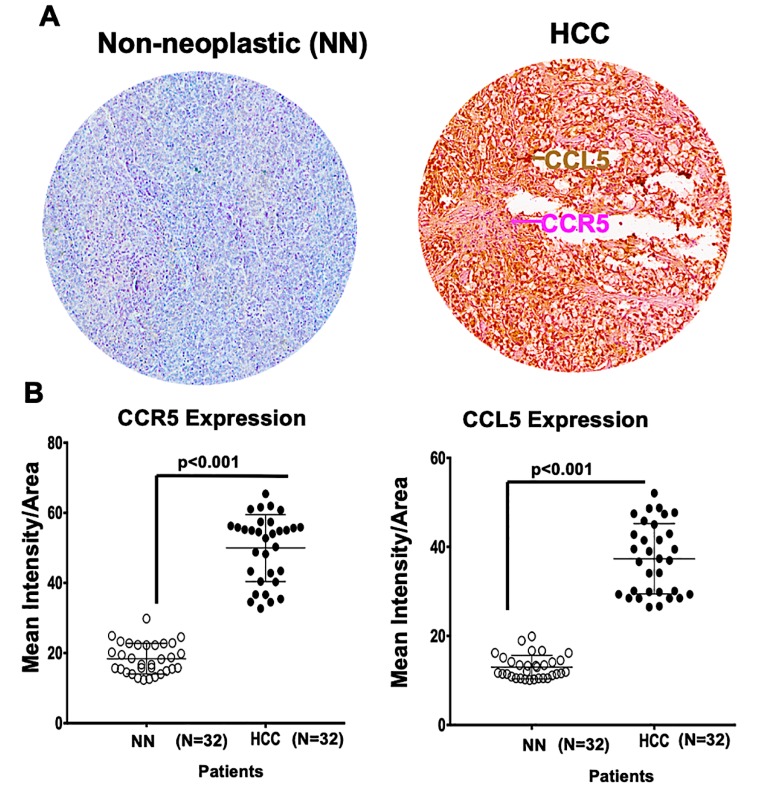
CCR5 and CCL5 are overexpressed in liver cancers (LCas) compared to adjacent tissues. (**A**) Liver tissues from non-neoplastic (NN) areas and hepatocellular carcinomas (HCCs) were stained with anti-CCL5 and anti-CCR5 antibodies. Magenta (AP) and brown (DAB) colors show CCR5 and CCL5 staining, respectively. A 40× objective was used to capture digital images from the slides. (**B**) Scatter diagrams were used to plot immunointensity scores determined by immunohistochemistry (IHC) of non-neoplastic tissues (*N* = 32) and HCCs (*N* = 32). Cells were categorized as to stain intensity 0 (blue), 1 + (yellow), 2 + (orange) and 3 + (Red). There were significant differences (*p* < 0.001) between HCCs and non-neoplastic tissues.

As clinicopathological nomograms are used to stratify risk in cancer, many recent novel technologies are used to understand tumor biology. However, immunohistochemistry analysis has been used as a robust tool in defining distinct cancer behaviors. In the present investigation, we examined, in a microarray, HCC (*n* = 32) and non-neoplastic (*n* = 32) tissues by staining for CCR5 and CCL5. Representative images in Figure 1A showed the protein expression of CCR5 and CCL5 in the tissues derived from HCC cancer patients and control samples. Using the scatter plot scores diagram, the immunointensity scores showed expression of both CCL5 and its receptor CCR5 that was three-fold higher in the HCC tissues (*p* ˂ 0.0001, Figure 1B) compared to the non-neoplastic ones. These clinical analyses suggest the importance of CCR5/CCL5 expression in determining the clinicopathological condition of HCC, and CCR5/CCL5 axis is involved in HCC progression.

### 2.2. Human-Derived HCC Cells Display Higher Levels of CCR5 Expression

**Figure 2:**
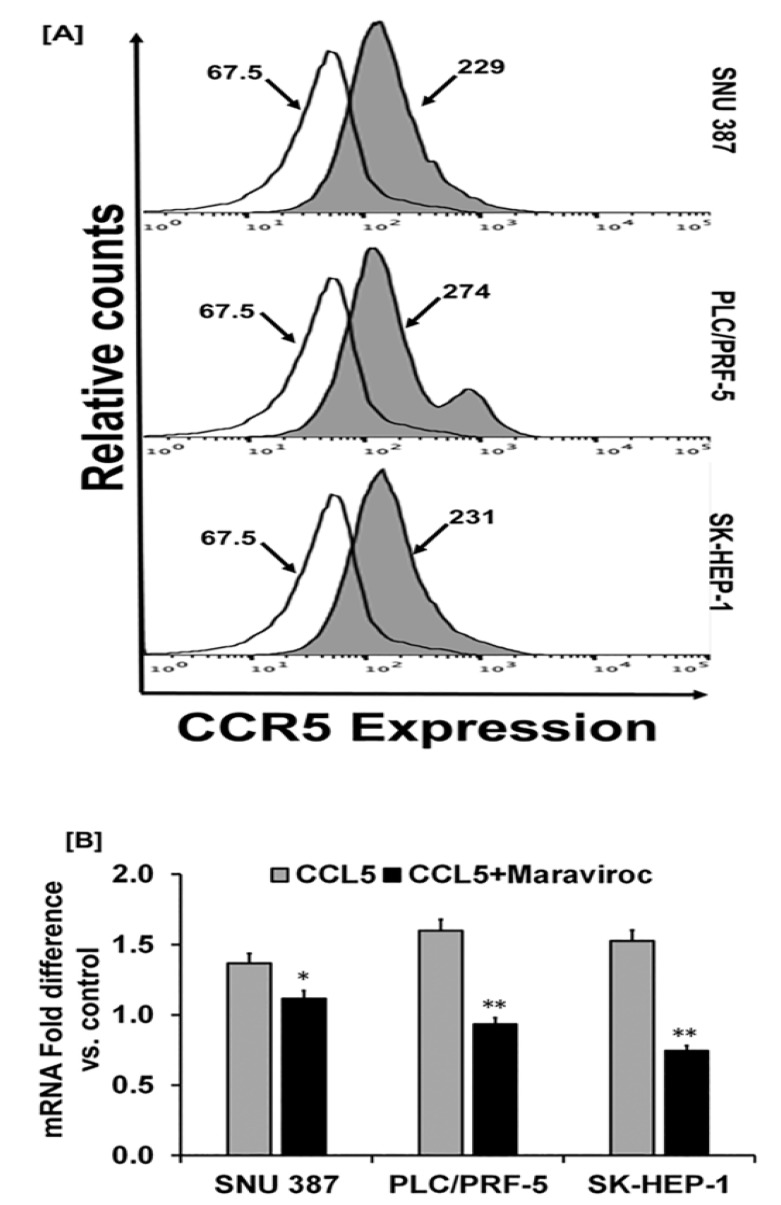
HCC cells display higher expression of CCR5. (**A**) Histograms presenting isotype control and respective CCR5 in HCC cell lines (SNU387, PLC/PRF-5, and SK-HEP-1). Cells were stained with a FITC-conjugated anti-CCR5 antibody or isotype. (**B**) Relative mRNA expression of CCR5 induced by CCL5 (100 ng/mL) with or without the inhibitor, maraviroc (200 ng/mL) for 30 min. To normalize the data, 18S rRNA was used as an endogenous control. The experiments were repeated three times. Bar graphs are presented as fold change in expression (±standard error); asterisks indicate *p* values (* *p* < 0.05 ** *p* < 0.01).

Utilizing flow cytometry analyses, the HCC cell lines, SNU387 (Asian), PLC/PRF-5 (Caucasian), and SK-HEP-1 (Caucasian), showed elevated expression of CCR5 (Figure 2A). For these cells, there was a demarcation between the control and the test samples in the upregulated expression of CCR5. The mean fluorescent values were 229, 274, and 231 for SNU387, PLC/PRF-5, and SK-HEP-1 cells, respectively. Furthermore, quantitative PCR revealed a high expression of CCR5 mRNA in the cells (Figure 2B), an observation similar to that determined by flow cytometry. In addition to the observation of CCR5 expression in human-derived HCC tissues, the cell lines that showed several-fold expressions of CCR5 in HCC cells indicate the importance of CCR5 in liver cancer.

### 2.3. CCL5 Enhances the CCR5 Expression in HCC Cell Lines

**Figure 3:**
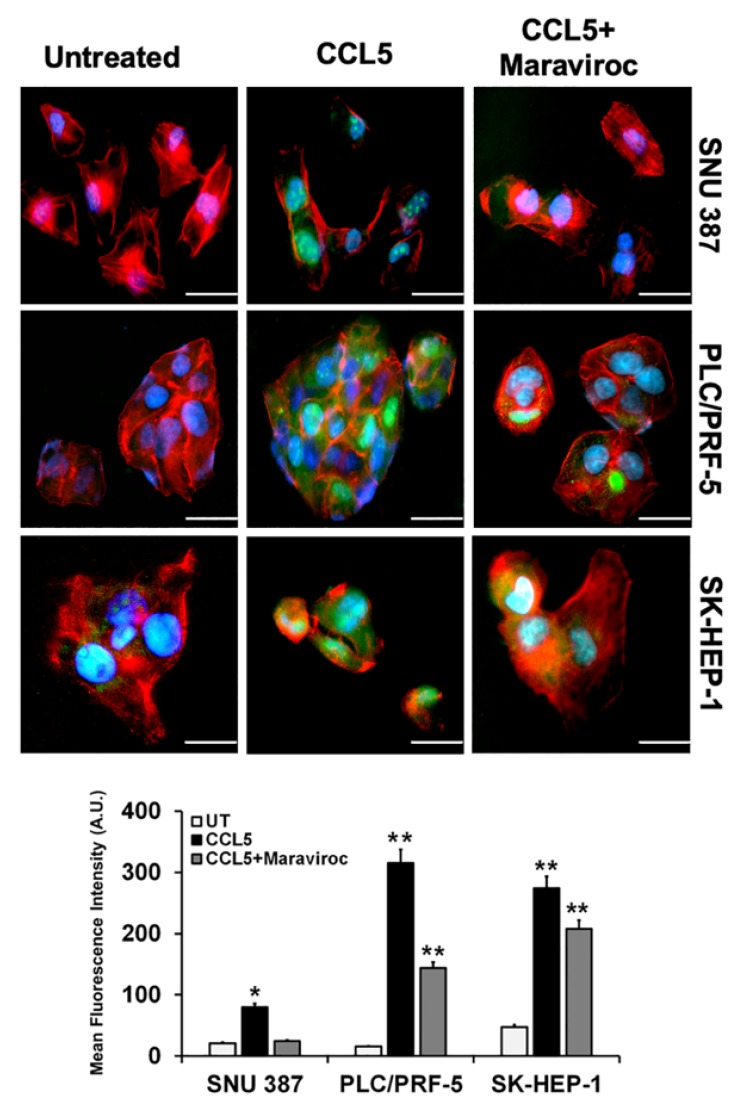
CCL5 induces CCR5 expression in HCC cells. (**A**) HCC cells were treated with CCL5 (100 ng/mL) and the CCR5 antagonist, maraviroc (200 ng/mL) for 30 min. FITC-conjugated anti- CCR5 antibody (green) and phalloidin (red) were used to detect CCR5 expression and the F-actin cytoskeleton. Nuclei were counterstained with DAPI (blue). Images were captured with a 40× objective. Scale bar = 100 µm. (**B**) Bar graph showing representative mean fluorescence intensities of CCR5 in cells treated with or without maraviroc. The asterisks indicate significance determined by Student’s *t*-test (* *p* < 0.05 ** *p* < 0.01).

**Figure 4:**
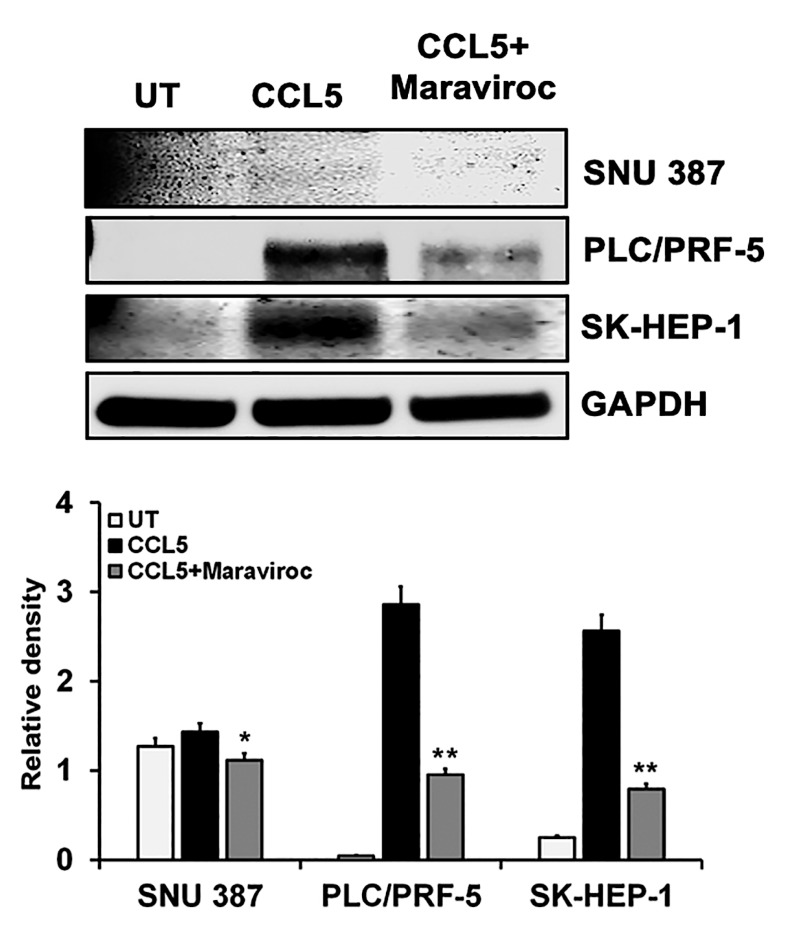
Western blot expression of CCR5 in HCC cells is induced by CCL5. HCC cells were treated with CCL5 with or without maraviroc for 30 min, and the expression of the CCR5 protein was analyzed by immunoblots. The house-keeping marker (GAPDH) was used as a loading control. The immunoblots shown are representative of three independent experiments. Densitometric evaluations of the proteins are shown in the lower panel. Data, presented as means (±standard error), were analyzed by Student’s *t*-test. ** and * indicate *p* values ≤ 0.01 and 0.05, respectively.

Since CCL5 induces the proliferation of cancer cells that express CCR5 [20], we evaluated the role of CCL5 in inducing CCR5 in HCC cells. For cells exposed to CCL5, quantitative real-time PCR (Figure 2B) showed a high expression of CCR5. To confirm the effect of CCL5 on the expression of CCR5 in HCCs, we used maraviroc (Sigma Aldrich, St. Louis, MO, USA), an antagonist of CCR5 that is well tolerated and is approved by the U.S. Food and Drug Administration [23]. Treatment of HCC cells with maraviroc decreased the expression of CCR5 at the mRNA level. In addition to the qPCR analysis, the effect of CCL5 on the expression of CCR5 was observed by cellular staining using immunofluorescence techniques that reflected protein levels. Similar observations were made through immunofluorescence staining of the cell lines, in which the expression of CCR5 was enhanced through the presence of CCL5 but was diminished in the presence of the antagonist (Figure 3). The results showed higher expression of CCR5 in PLC/PRF-5 and SK-HEP-1 cells upon CCL5 treatment compared to SNU387 cells. Furthermore, as shown by Western blots, protein levels of CCR5 were enhanced in the presence of CCL5 but were reduced by maraviroc (Figure 4). The protein band intensity/reading ratios are shown in Supplementary Figure S1. These findings indicate that the administration of maraviroc could reduce disease progression for HCCs.

### 2.4. CCR5/CCL5 Interaction Indicates the Migration Potential of HCC Cells by Enhancing the Expression of Akt and Epithelial to Mesenchymal Transition (EMT) Markers

**Figure 5:**
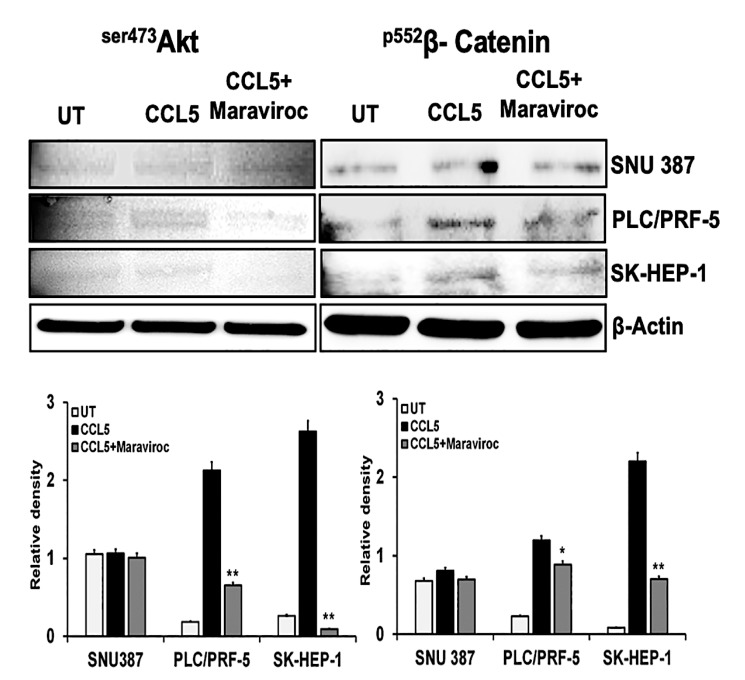
The CCL5/CCR5 interaction mediates cell survival and the epithelial to mesenchymal transition (EMT) of HCC cells. HCC cells were treated with CCL5 with or without maraviroc for 30 min, and an expression of the cell survival marker (^Ser473^Akt) and a marker for the EMT (^P552^β-catenin) were analyzed by immunoblots. β-Actin was used as a loading control. The immunoblots shown are representative of three independent experiments. Densitometric assessments of the proteins are shown in the lower panel. Data, presented as means (±standard error), were analyzed by Student’s *t*-test. ** and * indicate *p* values ≤ 0.01 and 0.05, respectively.

**Figure 6:**
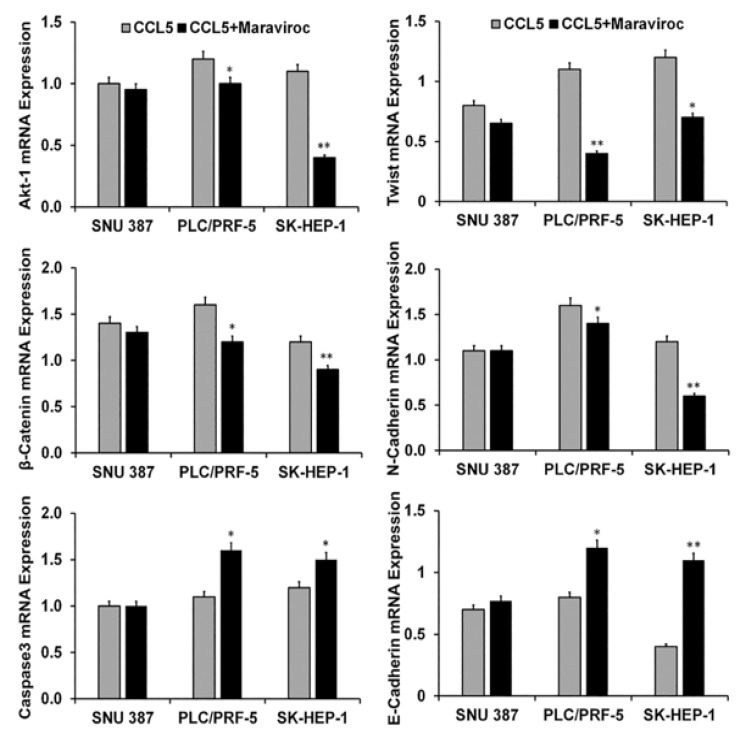
Effect of CCR5 overexpression or inhibition on cell survival, apoptosis, and the EMT in HCC cells. HCC cells were treated with CCL5 with or without maraviroc for 30 min. The bar graphs present the mRNA fold changes in expression of cell survival (Akt-1), apoptosis (caspase-3), and EMT (β- catenin, Twist, N-cadherin, and E-cadherin) markers. To normalize the data, 18S rRNA was used as an endogenous control. The experiments were repeated three times. Bar graphs are presented as fold change in expression (±standard error); the asterisks indicate *p* values (* *p* < 0.05 ** *p* < 0.01).

We determined the effect of CCL5 on the Akt pathway in HCC cells. Compared to controls, protein expression of ^ser473^Akt was high in PLC/PRF-5 and SK-HEP-1 cells treated with CCL5 but were minimal in SNU387 cells (Figure 5). Next, we determined if the CCR5/CCL5 interaction was involved in the activation of the EMT. For this, we used β-catenin as a marker of the EMT. Similar to the activation of the PI3K/Akt pathway, there was, for two-cell lines, a high expression of β-catenin (Figure 5), which was diminished by maraviroc. The protein band intensity/reading ratios are shown in Supplementary Figure S2. Similar results were obtained at the mRNA transcript levels, which showed Akt-1 expression levels of nearly 1.3- and 1.2-fold in PLC/PRF-5 and SK-HEP-1 cells, respectively (Figure 6). Similarly, there was a 1.7- and 1.3-fold increase of β catenin in PLC/PRF-5 and SK-HEP-1 cells while these markers (Akt-1) remained unchanged in SNU387. To establish that these effects are due to the CCL5 and CCR5 interaction, the cells were treated with the CCR5 inhibitor. The levels of the Akt-1 and β-catenin were diminished by maraviroc, indicating the involvement of the CCR5/CCL5 interaction in the activation of the PI3K/Akt pathway. In addition, after exposure of the cells to maraviroc, there was a high expression of E-cadherin (0.3 and 0.8 fold) in PLC/PRF-5 and SK-HEP-1 cell lines, respectively, while there was decreased in expressions of Twist and N-cadherin compared to CCL5 treated cells. Overall, we observed that the use of maraviroc can regulate the EMT markers while abrogating the cell survival and progression of HCC cells at the transcriptional level. 

### 2.5. Inhibition of the CCR5/CCL5 Interaction Leads to Apoptosis of HCC Cells

Since stimulation of the CCR5/CCL5 interaction induces the proliferation of various cancer cells [20], we determined if inhibition of the interaction would lead to apoptosis of HCC cells. With caspase-3 as an indicator for apoptosis, we measured its mRNA levels in SNU387, PLC/PFR-5, and SK-HEP-1 cells. In the presence of CCL5, the transcript levels of caspase-3 were down-regulated (Figure 6). However, with inhibition of the interaction between CCR5 and CCL5, higher levels of caspase-3 mRNA were present in two of the three cell lines where it had increased nearly 0.5-fold. This indicated that the CCR5/CCL5 interaction was involved in promoting the proliferation of HCC cells. 

### 2.6. The CCR5/ CCL5 Interaction Induces Metastatic Behavior in HCC Cells

**Figure 7:**
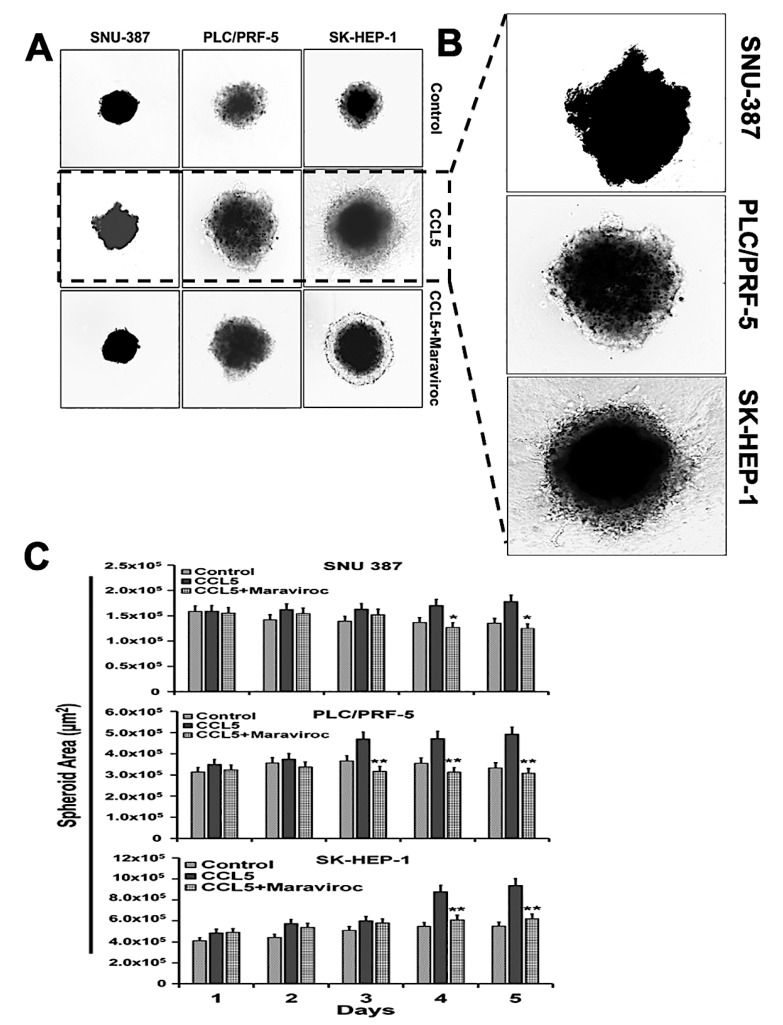
Treatment with the CCR5 antagonist maraviroc blocks the migration and invasion of HCC cells. (**A**) Three-dimensional morphology of invading cells over a 5-days for HCC cells untreated and treated with maraviroc. (**B**) Representative images showing CCL5-induced migration and invasion of liver cancer cells. (**C**) Quantitative surface area analysis of HCC cells over 5-days. The experiments were repeated three times. Bar graphs are presented as a change in surface area in expression (±standard error); the asterisks indicate *p* values (* *p* < 0.05 ** *p* < 0.01).

Since the CCR5/CCL5 interaction is involved in the motility, migration, and invasion of various cancer cells [18,24,25], we analyzed the functional significance of this interaction in the migration of HCC cells. Three-dimensional cultures of SNU387, PLC/PFR-5, and SK-HEP-1 cells exposed to CCL5 were used (Figure 7). With the addition of CCL5 to the medium, the invading cells moved out of the spheroids into the surrounding matrix with spindle-like projections, indicating their migratory potential. To show that the migration and invasive potential of the cells was due to the CCR5/CCL5 interaction, the cells were treated with the CCR5 inhibitor, maraviroc. The migratory potential of the cells was reduced. These observations confirmed the involvement of the CCR5/CCL5 interaction in the metastatic potential of HCC cells.

## 3. Discussion

HCC, with an increasing yearly incidence, is the fourth most common cause of cancer-related deaths worldwide [26]. HCC is characterized by its aggressiveness, poor response to conventional treatments, and a low survival rate [27]. Identification of the molecular events associated with HCC will allow a better understanding of its pathogenesis and could lead to the development of new therapeutic strategies [27]. The chemokine/receptor axis, along with events associated with the immune system [28], is a factor in the progression of various cancers.

In the present investigation, analysis of several human HCC cell lines found enhanced expression of CCR5 and CCL5 relative to normal tissues. CCL5 is produced by cancer cells and by non-malignant stromal cells at primary or metastatic sites [29]. Our histological analysis of primary tumors showed even distributions of CCR5 and CCL5 in tissue sections of HCCs. Although the functions of CCR5 and CCL5 in the HCC tissues are unknown, the CCR5/CCL5 axis correlates with chronic inflammation of the liver that is induced by various pathogens, and it participates in the development of HCCs [30,31]. Since, as seen in this report, the human-derived HCC tissues showed over-expression of CCR5 and CCL5, these could function as prognostic markers for HCC.

CCR5 expression is implicated in the growth of various cancers, including breast cancer, ovarian cancer, cervical cancer, prostate cancer, colon cancer, melanoma, Hodgkin’s lymphoma, and multiple myeloma [20]. Cancer cells secrete CCL5 or they induce adjacent fibroblasts to secrete CCL5, which ensures the proliferation of CCR5-positive cells [20]. In the present investigation, flow cytometry analysis of HCC cell lines showed high expression of CCR5. That fact that CCL5 stabilizes the expression of CCR5 was evident by the increased expression levels of CCR5 in the presence of CCL5. However, the CCR5 levels were low after HCC cells were treated with the CCR5 inhibitor, maraviroc. These observations support the view that CCL5 sustains the proliferation of HCC cells.

The PI3K/Akt/mTOR pathway is implicated in the development of HCCs [27]. Although for various cancers, overexpression of efflux pumps is often associated with chemoresistance, deregulation of the PI3K/Akt/mTOR pathway contributes to the resistance of HCCs to drugs acting on microtubules, including vincristine, colchicine, and paclitaxel [32,33]. Various growth factors and cytokines stimulate the activation of receptor tyrosine kinases and G protein-coupled receptors. During this activation, PI3K is recruited to the membrane by direct or indirect interaction with the receptors [34]. Activated PI3K, with its secondary messengers, activates the Akt pathway. Akt, a downstream effector of the PI3K pathway, includes three isoforms, Akt1, Akt2, and Akt3, which activate downstream signaling effectors to regulate angiogenesis; cell cycle progression and cell survival, proliferation, and migration [35]. As such, this pathway is a potential therapeutic target for anticancer therapy. The present study showed that the CCR5/CCL5 interaction was responsible for activation of the Akt pathway, an effect that was reversed by the CCR5 antagonist, maraviroc.

In the EMT, hepatic epithelial cells lose their adhesive properties, attain a mesenchymal nature, and gain invasive/metastatic capability [36]. The CCR5/CCL5 interaction is involved in the metastasis of various cancer cells [18]. In metastatic LCa cells, the epithelial marker, E-cadherin, is downregulated, and mesenchymal markers, such as vimentin and N-cadherin, are upregulated. In other cancers, the CCL5/CCR5 interaction deregulates β-catenin, which stimulates the invasiveness of cancer cells [37]. In the present study, we found similar deregulation of β-catenin expression. We also found that the interaction of CCR5 and CCL5 diminished the expression of E-cadherin, an effect that was reversed by the CCL5 inhibitor. These observations show that the CCR5/CCL5 interaction regulates the EMT transcriptional factors and other proteins, making the HCC cells metastatic and invasive.

As shown in our studies, the varying levels of CCR5 expression in the presence and absence of CCL5 determined the role of the CCR5/CCL5 axis in promoting tumor growth and progression; this was supported by the tissue microarray data. However, the SNU387 cell line showed no significant changes in CCR5 expression even in the presence of the CCL5 ligand. Studies of the role of chemokines and their receptors in the development of LCa disparities show that genetic and geographical factors may be involved [38]. The CCR5 delta 32 allele (a 32-bp deletion gene mutation of the CCR5 gene), a nonfunctional form of the receptor, is implicated in various immune-related functions [39]. This became evident in determining the role of CCR5 in immune responses in an Iranian population between healthy and HBV-infected individuals. CCR5Δ32 is more frequent in healthy individuals [39]. CCR5Δ32 is not present in populations of Southeast Asian countries and China [39], but CCR5Δ32 heterozygosity is present in the Indian population [38,40]. We hypothesize that the weak CCR5/CCL5 interaction in the SNU387 cell line (Asian) could be due to a delta32 mutation. However, sequencing studies are needed to reveal the reasons for lower CCR5 expression.

**Figure 8:**
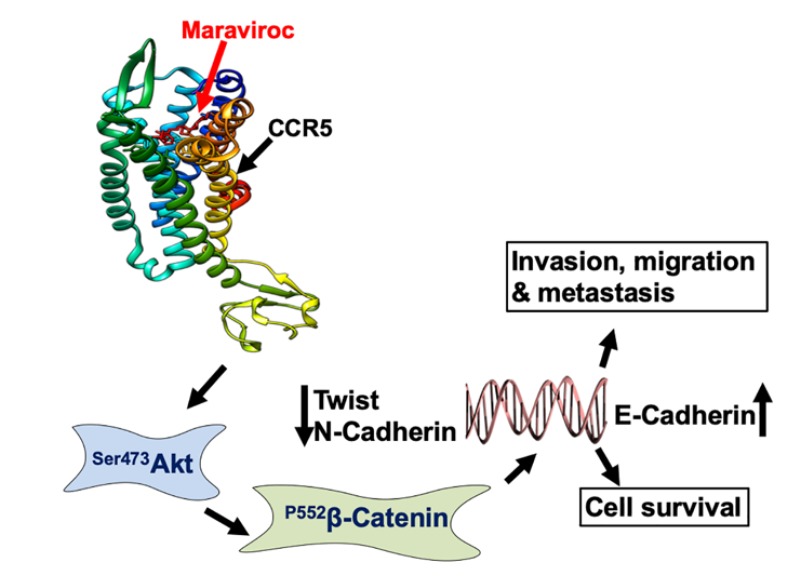
Schematic representation of the CCR5/CCL5 interaction inducing cell survival, invasion, migration, and metastasis. Crystal structure (PDB id: 4mbps) depicting a CCR5/CCL5 interaction that activates the Akt-1 downstream pathway to promote the migration and invasion of LCa cells.

CCR5 is present in both immune cells and cancer cells, and it has dual roles, both anti-tumor and tumor-promoting [41,42]. Although the function of CCR5 remains controversial, perhaps based on the cell type in which it is expressed, recent data indicate that CCR5 mobilizes cancer cells with its tumor-promoting activity [43]. However, inhibition of CCR5 with the inhibitor, maraviroc, led to a positive outcome for cancer patients [23]. In the present study, we observed that maraviroc, reduced the growth of HCC cells and induced apoptosis, as indicated by elevated expression of caspase-3. A schematic presentation of the CCR5/CCL5 interaction inducing cell survival, invasion, migration, and metastasis is shown in Figure 8. These results indicate that the CCR5/CCL5 axis is a potential target for the treatment of HCCs.

## 4. Materials and Methods

### 4.1. Cell Lines and Cultures

The human LCa cell lines, SNU387 (ATCC^®^ CRL-2237), PLC/PRF-5 (ATCC^®^ CRL-8024), and SK-HEP-1 (ATCC^®^ HTB-52), were purchased from American Tissue Culture Collection (ATCC, Manassas, VA, USA). SNU387 cells were cultured in RPMI-1640 media; PLC/PRF-5 and SK-HEP-1 cells were grown in EMEM supplemented with 10 % fetal bovine serum (FBS), 10,000 U/mL penicillin, and 10,000 μg/mL of streptomycin (Fisher Scientific, Pittsburg, PA, USA). All cell lines were maintained in a humidified incubator containing 5% CO_2_ at 37 °C.

### 4.2. Immunohistochemistry (IHC) of Clinical Samples 

Human liver tissue microarray slides were obtained from AccuMax Array Inc. (ISU Abxis Co. Plaisir, France) (demographic information shown in Table S1). Following our previous protocol [18], IHC of LCa markers was conducted for 64 tumor tissues. Briefly, slides with tissue specimens were dewaxed in xylene and rehydrated in a graded alcohol series (100%, 95%, and 70%, 5 min each) (Fisher Scientific, Pittsburg, PA, USA). Antigen retrieval (pH 8.1) (Biolegend, San Diego, CA, USA) was performed for 30 min, and endogenous peroxidase activity was blocked by 3% H_2_O_2_. The sections were washed with phosphate-buffered saline-T (PBS + 0.05% Tween 20) and were blocked with normal donkey serum (5%, Jackson Immuno Research, PA, USA) for 1 h. Following our previous method, the sections were incubated with primary antibody for CCL5 (10 μg/mL, R & D systems, Minneapolis, MN, USA) at 4 °C overnight and with secondary antibody for 1 h at room temperature, and then incubated with streptavidin-horseradish peroxidase (HRP) (Biolegend, San Diego, CA, USA), stained, and developed with 3′3-diaminobenzidine (DAB, Biolegend, San Diego, CA, USA). Further, sections were washed again with PBS-T and incubated with primary antibody anti-CCR5 (10 μg/mL) (R & D systems, Minneapolis, MN, USA) for 3 h and then with secondary antibody. They were subsequently washed, incubated with streptavidin-alkaline phosphatase (Jackson Immuno Research, PA, USA), and developed in alkaline phosphatase red chromogen coloring agent for detection of CCR5. Finally, the nuclei were counterstained with hematoxylin (Fisher Scientific, Pittsburg, PA, USA); and slides were dehydrated, cleared, and mounted. Digital images were captured with a 40× objective (Histowiz Inc., Brooklyn, NY, USA) and analyzed using an Aperio ImageScope v. 6.25 software (Aperio Technologies, USA). The positive pixel counts of CCR5 and CCL5 intensities were quantified using algorithms provided by Aperio ImageScope v. 6.25 software. The results were displayed in dot-plot diagrams. Cut-offs (to differentiate between positive and negative cells) and gates (to accentuate between cell populations) were set to derive data for the dot blots.

### 4.3. Flow Cytometry

To assess the expression of CCR5, SNU387, PLC/PRF-5, and SK-HEP-1 cells were seeded in 6-well plates and cultured overnight. The cells were trypsinized (0.05% trypsin), harvested, and washed with PBS supplemented with 2% fetal bovine serum (FACS buffer, Fisher Scientific, Pittsburgh, PA, USA) and counted. Further, cells were blocked with Fc Block (BD Bioscience, CA, USA) and stained with FITC-conjugated mouse IgG2a (4 µL, Biolegend, San Diego, CA, USA) isotype control or anti-human CCR5 antibody (5 µL per 100 µL) (R & D systems, Minneapolis, MN, USA) FACS buffer for 30 min. Finally, cells were washed, suspended in FACS buffer, and analyzed by flow cytometry using Guava easyCyte HT (EMD Millipore, Billerica, MA, USA).

### 4.4. Immunofluorescence

Cells were cultured in 48-well plates overnight, washed with PBS, and placed in serum-free media for 12 h at 37 °C in a humid atmosphere of 5% CO_2_. Next, cells were treated with CCL5 (100 ng/mL) (Biolegend, San Diego, CA, USA) recombinant protein for 30 min. For each of the three liver cell lines, maraviroc (200 ng/mL, Sigma Aldrich, St. Louis, MO, USA), was added to block the activity of CCL5. Further, cells were washed, fixed with 4% paraformaldehyde, and permeabilized by saponin (Fisher Scientific, Pittsburg, PA, USA) for 10 min at 4 °C. Cells were washed with PBS, blocked with 3% bovine serum albumin (Sigma Aldrich, St. Louis, MO, USA) for 1 h, and then stained with FITC-conjugated anti-CCR5 antibody (Biolegend, San Diego, CA, USA) overnight at 4 °C. F-Actin filaments were stained with Phalloidin Red 594 solution (1:40) (Biolegend, San Diego, CA, USA) for 20 min at room temperature. Lastly, nuclei were counterstained with DAPI (Invitrogen, Carlsbad, CA, USA), and images were captured at 40× using a fluorescent microscope (EVOS FL microscope; Thermo Scientific, USA).

### 4.5. Western Blot Analysis

To determine protein expression, cells were grown overnight and treated with CCL5 (100 ng/mL) (Biolegend, San Diego, CA, USA) with or without an inhibitor, maraviroc (200 ng/mL, Sigma Aldrich, St. Louis, MO, USA). Cell lysis was accomplished by using Radioimmuno Assay buffer (RIPA) (Thermo Scientific, Rockford, IL, USA) at 4 °C, and the concentrations of proteins were determined with bicinchoninic acid protein assay kits (Thermo Scientific, Rockford, IL, USA). The cell lysates were mixed, and proteins were denatured with Laemmli buffer (Fisher Scientific, Pittsburg, PA, USA) for 10 min at 95 °C. Equal amounts of protein (30 µg) were separated by 4–12% sodium dodecyl sulfate-polyacrylamide gel electrophoresis (SDS-PAGE) and then transferred electrophoretically to nitrocellulose membranes by iBlot dry blotting (Thermo Scientific Rockford, IL, USA). The membranes were blocked with 5% non-fat dry milk (Bio-Rad, Hercules, CA, USA) prepared in TBS-T (20 mM TRIS-HCL, pH 7.6; 150 mM NaCl) (Fisher Scientific, Pittsburg, PA, USA) containing 0.1% Tween-20 for 1 h. For the detection of CCR5, the membranes were incubated with a primary antibody, anti-CCR5 (R & D systems, Minneapolis, MN, USA) overnight at 4 °C. Antibodies for Akt phospho (Ser473) (Biolegend, San Diego, CA, USA) and β-catenin were procured from Cell Signaling Technology, Danvers, MA, US. The primary antibodies were diluted in 5% non-fat milk containing a TBS-T buffer. The membranes were incubated with HRP-conjugated antibodies, anti-mouse and/or anti-rabbit (1:2000), for 2 h at room temperature, followed by washing with PBS-T three times. To ensure equal loading, GAPDH or β-actin (1:1000) (Cell Signaling Technology, Danvers, MA, USA) was used as an internal control. Finally, immunoblots were processed with ECL Prime Western blotting chemiluminescent detection reagent (GE Healthcare-Biosciences, Pittsburgh, PA, USA), and immunoreactive proteins were visualized by Image Quant LAS4000 (GE Healthcare-Biosciences, Pittsburgh, PA, USA). The band intensities were quantified by use of the Image-J software (NIH).

### 4.6. Quantitative Reverse Transcription-Polymerase Chain Reaction (qRT-PCR)

Following steps in our previous publication [18], cells were grown, harvested, and lysed with Trizol reagent (Invitrogen, Paisley, UK). The total RNA was converted to cDNA using reverse transcription super mix for RT-qPCR (Bio-Rad, Hercules, CA, USA) according to the manufacturer’s instructions. All primer sequences were synthesized from data in the National Center for Biotechnology Information (NCBI) gene bank database. The sequences of primers for 18S, CCR5, Akt-1, β-catenin, caspase-3, Twist, N-cadherin, and E-cadherin are shown in Table 1. RT-PCR was performed by using SYBR^®^ Green PCR master mix reagents (Bio-Rad, Hercules, CA, USA), and gene expression was analyzed by CFX-Manager software (CFX96 Real-Time System, Bio-Rad, Hercules, CA, USA). As an endogenous control, 18S rRNA was used. Fold changes were calculated using relative quantification, and the experiments were repeated three times.

### 4.7. 3 D Cell Migration and Invasion Assay

Three-dimensional cell cultures are a tool for drug screening and for evaluating the invasion and migratory potential of cells [18,44]. To develop 3D cell spheroids, SNU387, PLC/PFR-5, and SK-HEP-1 cells were incubated overnight with an appropriate media and a magnetic gold–polymer–iron oxide hydrogel (Nano shuttle-PL, n3D Biosciences, Houston TX, USA) to allow the nano-shuttle to attach to the cells [45]. Next, cells were washed, trypsinized, and counted with a hemocytometer. Cells (4 × 10^3^) were seeded in 24-well cell-repellent plates, where they were magnetically levitated off the bottom to form a 3D structure in 3–4 h. Further, 3D cell structures were placed on a drive of magnets for 15 min to make spheroids by following the manufacturer’s instructions (n3D Biosciences, Houston TX, USA). Following our previous protocol [18] involving an invasion matrix (Cultrex96 well 3D Spheroid BME Cell Invasion Assay kits (Amsbio, MA, USA)), 3D spheroids were treated with CCL5 (100 ng/mL Biolegend, San Diego, CA, USA) with or without maraviroc (200 ng/mL). Cells were incubated for 5 days and maintained in a humidified incubator containing 5% CO_2_ at 37 °C. Every 24 h, images were taken microscopically with a 10× objective. Changes in the 3D spheroid area were analyzed using Image-J software (NIH).

### 4.8. Statistical Analysis

All measurement data were presented as standard errors of means (±SEM) for at least three independent experiments. For comparison across groups, one-way analysis of means (ANOVA) was applied followed by Tukey’s post hoc test. For comparison between groups, a two-sample t-test was applied. In addition, for comparison within groups, paired *t*-tests were used. Intensities of the receptor (CCR5) and the ligand (CCL5) were examined for normality assumptions using the Shapiro–Wilk test and transformed to a log scale. Stat view II programs (Abacus Concepts, Inc., Berkeley, CA, USA) were used to analyze the data and were considered statistically significant if *p* values < 0.05. With FlowJo Software, the Kolmogorov–Smirnov two-sample test was used to compare the histograms.

## 5. Conclusions

The involvement of chemokines in various cancers and their stimulation of the chemokine receptors expressed in cancer cells have established them as regulators of biological processes. In the present study, we showed that CCR5 and CCL5 could serve as prognostic markers for human HCCs. Through studies of HCC cell lines, we found that CCL5 stimulated the expression of CCR5. Further, we showed that this interaction led to growth, proliferation, and migration of HCC cells, effects that were reversed by the CCR5 antagonist, maraviroc. Finally, we demonstrated that blockade of the CCR5/CCL5 interaction could induce apoptosis of these cells. These findings establish that the CCR5/CCL5 interaction is a target for managing and treating HCC in humans.

## Figures and Tables

**Table 1:** Primer sequences used for qRT-PCR.

Gene	Sense	Antisense
*18S*	5′-GGCCCTGTAATTGGAATGAGTC-3′	5′-CCAAGATCCAACTACGAGCTT-3′
*CCR5*	5′-GCAAGGAGACCACCAACAG-3X	5′-CCCTCACTTCCAACCCAAATC-3′
*Akt-1*	5′-ATGGACAGGGAGAGCAAACG-3′	5′-CTGGCCACAGCCTCTGATG-3′
*β-catenin*	5′-TCCTCAGATGGTGTCTGCTA-3′	5′-GATGATGGGAAAGGTTATGC-3′
*Caspase-3*	5′-CTCTGGTTTTCGGTGGGTGT-3′	5′-CGCTTCCATGTATGATCTTTGGTT-3′
*Twist*	5′-AGCTGAGCAAGATTCAGACC-3′	5′-CAGCTTGCCATCTTGGAGT-3′
*N-cadherin*	5′-TACAGACATGGAAGGAATCCCC-3X	5′-ATGGCAGTAAACTCTGGAGGA-3′
*E-cadherin*	5′-CGTCCTGGGCAGAGTGAAT-3′	5′-TTTGAATCGGGTGTCGAGGG-3′

## Supplementary Materials

The following are available online at https://www.mdpi.com/2072-6694/12/4/883/s1, All Western blot densitometry readings/intensity ratios tables and Figure S1: Western blots supplementary figures showing the expression of CCR5 makers in liver cancer cells, Figure S2: Western blots supplementary figures showing the expression of p-Akt and β-Catenin makers in liver cancer cells are available in supplementary materials. Pathological information for liver tissues is in Table S1: Demographic information of human liver cancer tissue.
