# Supplementary material for: Biological and Clinical Significance of the CCR5/CCL5 Axis in Hepatocellular Carcinoma
Source: Cancers (Basel). 2020 Apr 5;12(4):883. doi: 10.3390/cancers12040883 (PMC7226629; doi:10.3390/cancers12040883)
Supplement: Supplementary file 1 [file cancers-12-00883-s001.pdf]

cancers

MDPI

Article

Biological and Clinical Significance of the CCR5/CCL5 Axis in Hepatocellular Carcinoma

Santosh K. Singh, Manoj K. Mishra, Brian M. Rivers, Jennifer B. Gordetsky, Sejong Bae and Rajesh Singh

Supplementary Materials 1.

(A)

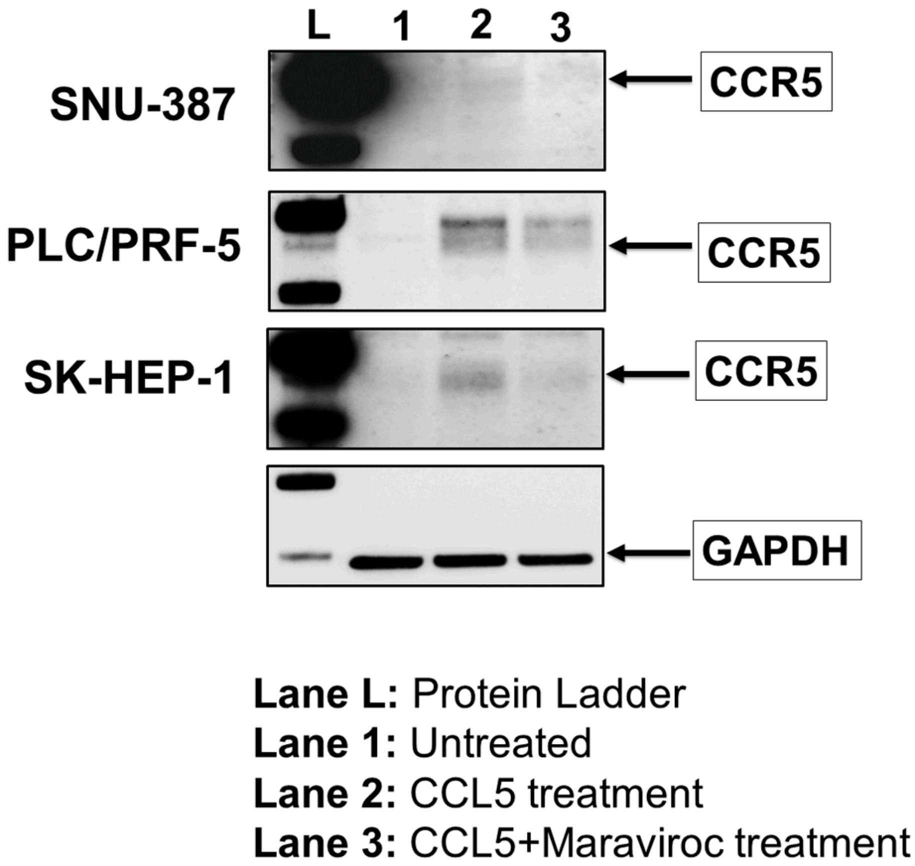

(B) Densitometry readings/intensity ratio.

	CCR5/GAPDH		
	**SNU387**	**PLC/PRF-5**	**SK-HEP-1**
**UT**	1.269129	0.045158	0.250225
**CCL5**	1.43037	2.861028	2.56223
**CCL5+maraviroc**	1.112502	0.9526	0.793331

Figure S1. Western blots supplementary figures showing the expression of CCR5 makers in liver cancer cells. **(A)** HCC cells were treated with CCL5 with or without inhibitor (maraviroc) for 30 min and the expression of protein CCR5 (size 40kd) was analyzed by immunoblots. The house keeping marker GAPDH (37kD) was used as a loading control. For SNU-387, PLC/PRF-5 and SK-HEP-1 cells: Lane 1, 2, and 3 represent untreated, CCL5 treated and CCL5 with maraviroc treatment. L: represents the protein ladder. Images were cropped in the respective figures. (**B**) Densitometric readings/intensity ratio of Western blots for CCR5 in HCC cells. The house-keeping marker (GAPDH)

was used as a loading control. The ratios for immunoblot densitometry shown are representative of three independent experiments.

(A)

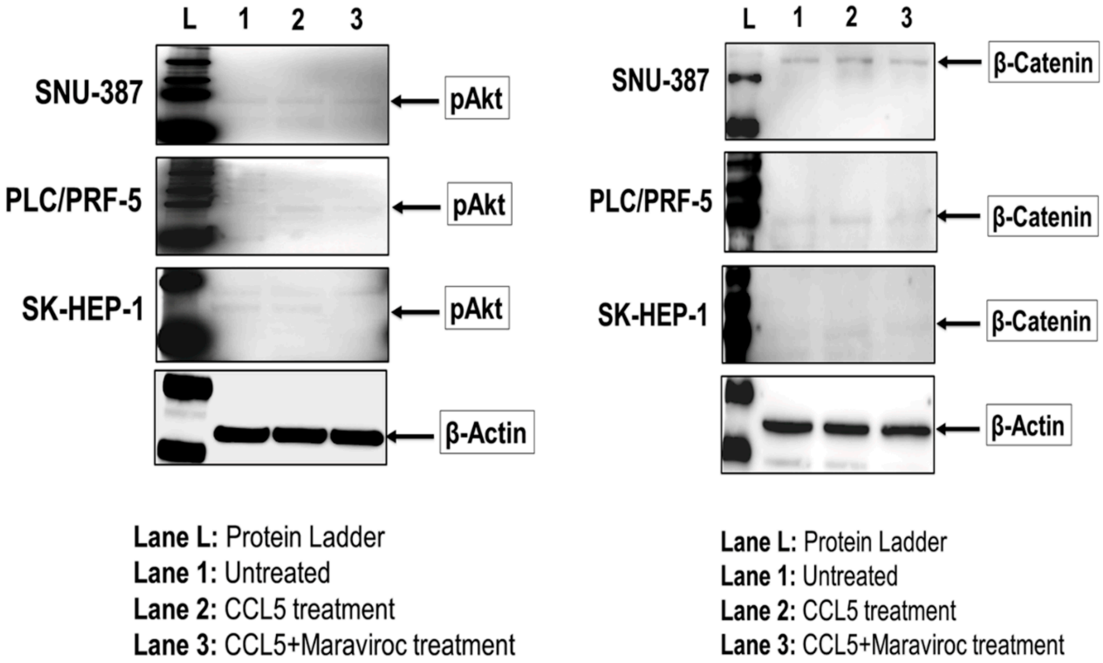

(B) Densitometry readings/intensity ratios.

	p-Akt/β-Actin		
	**SNU387**	**PLC/PRF-5**	**SK-HEP-1**
**UT**	1.052182	0.1866	0.261754
**CCL5**	1.059205	2.126713	2.627383
**CCL5+maraviroc**	1.009444	0.653153	0.090918

	β- Catenin/β-Actin		
	**SNU387**	**PLC/PRF-5**	**SK-HEP-1**
**UT**	0.680491	0.225679	0.082592
**CCL5**	0.809048	1.189804	2.198163
**CCL5+maraviroc**	0.698827	0.887205	0.702361

Figure S2. Western blots supplementary figures showing the expression of p-Akt and β-catenin makers in liver cancer cells. (**A**) HCC cells were treated with CCL5 with or without inhibitor (maraviroc) for 30 min and the expression of protein pAkt (60 kd) and β-catenin (92 Kd) was analyzed by immunoblots. The house keeping marker β-actin (45 kD) was used as a loading control. For SNU-387, PLC/PRF-5 and SK-HEP-1 cells: Lane 1, 2, and 3 represent untreated, CCL5 treated and CCL5 with maraviroc treatment. L: represents the protein ladder. Images were cropped in the respective figures. (**B**) Densitometric readings/intensity ratio of Western blots for Akt and β-catenin in HCC cells. The house-keeping marker (β-actin) was used as a loading control. The ratios for immunoblot densitometry shown are representative of three independent experiments.

Table S1. Demographic information of human liver cancer tissue.

No.	Sex	Age	Key word	Tumor grades
1	Female	73	Hepatocellular carcinoma	III
2	Female	73	Non-neoplastic	
3	Female	58	Hepatocellular carcinoma	II
4	Female	58	Non-neoplastic	
5	Male	81	Hepatocellular carcinoma	II
6	Male	81	Non-neoplastic	
7	Male	72	Hepatocellular carcinoma	II
8	Male	72	Non-neoplastic	
9	Male	80	Hepatocellular carcinoma	III
10	Male	80	Non-neoplastic	
11	Male	10	Hepatocellular carcinoma	II
12	Male	10	Non-neoplastic	
13	Male	88	Hepatocellular carcinoma	II
14	Male	88	Non-neoplastic	
15	Male	76	Hepatocellular carcinoma	III
16	Male	77	Non-neoplastic	
17	Male	59	Hepatocellular carcinoma	I
18	Male	59	Non-neoplastic	
19	Male	63	Hepatocellular carcinoma	III
20	Male	63	Non-neoplastic	
21	Male	57	Hepatocellular carcinoma	II
22	Male	57	Non-neoplastic	
23	Male	61	Hepatocellular carcinoma	I
24	Male	61	Non-neoplastic	
25	Male	65	Hepatocellular carcinoma	II
26	Male	65	Non-neoplastic	
27	Male	59	Hepatocellular carcinoma	III
28	Male	59	Non-neoplastic	
29	Female	66	Hepatocellular carcinoma	III
30	Female	66	Non-neoplastic	
31	Female	64	Hepatocellular carcinoma	III
32	Female	64	Non-neoplastic	
33	Male	80	Hepatocellular carcinoma	III
34	Male	80	Non-neoplastic	
35	Male	44	Hepatocellular carcinoma	III
36	Male	44	Non-neoplastic	
37	Male	56	Hepatocellular carcinoma	II
38	Male	56	Non-neoplastic	
39	Male	41	Hepatocellular carcinoma	III
40	Male	41	Non-neoplastic	
41	Male	55	Hepatocellular carcinoma	II
42	Male	55	Non-neoplastic	
43	Female	51	Hepatocellular carcinoma	III
44	Female	51	Non-neoplastic	
45	Male	63	Hepatocellular carcinoma	II
46	Male	63	Non-neoplastic	
47	Male	67	Hepatocellular carcinoma	II
48	Male	67	Non-neoplastic	
49	Male	73	Hepatocellular carcinoma	I
50	Male	73	Non-neoplastic	
51	Male	68	Hepatocellular carcinoma	I
52	Male	68	Non-neoplastic	
53	Male	66	Hepatocellular carcinoma	III
54	Male	66	Non-neoplastic	
55	Female	64	Hepatocellular carcinoma	III
56	Female	64	Non-neoplastic	
57	Male	56	Hepatocellular carcinoma	III
58	Male	56	Non-neoplastic	
59	Female	51	Hepatocellular carcinoma	II
60	Female	51	Non-neoplastic	
61	Male	42	Hepatocellular carcinoma	III
62	Male	42	Non-neoplastic	

63	Male	37	Hepatocellular carcinoma	III
64	Male	37	Non-neoplastic	

© 2020 by the authors. Licensee MDPI, Basel, Switzerland. This article is an open access article distributed under the terms and conditions of the Creative Commons Attribution (CC BY) license (http://creativecommons.org/licenses/by/4.0/).
